# Do Social Timing and Gender Matter to Parental Depression Aroused by Traumatic Experience of Child Bereavement? Evidence from China

**DOI:** 10.3390/ijerph182212058

**Published:** 2021-11-17

**Authors:** Dan Chen, Yuying Tong

**Affiliations:** Department of Sociology, The Chinese University of Hong Kong, Hong Kong 999077, China; yytong@cuhk.edu.hk

**Keywords:** child loss, social timing, son preference, psychological wellbeing, ageing and the life course perspective, intergenerational solidarity

## Abstract

Child loss is a rare but traumatic life event that often has a detrimental effect on parental wellbeing. However, parents’ resources and strategies in coping with the stressful child bereavement event may depend on timing of the event. This study intends to examine how parental depression could be aroused by the occurrence and timing of child bereavement, and how the influences vary by child gender. Drawing on the theoretical framework of the stress and life course, and using three waves of data from the China Health and Retirement Longitudinal Study, we find that both the occurrence and timing of child bereavement are significantly associated with parental depression in later life. Bereaved parents are more likely to report depression than non-bereaved parents. Child bereavement in children’s young adulthood is more likely to spark off parental depression than that occurring in children’s midlife or later. Further analysis confirms that the timing effect of child bereavement differs by child gender. Parents whose son died during young adulthood are more likely to report depression than their counterparts whose daughter died. Future studies need to address how to build up a specific social welfare program targeting child bereavement groups in different life stages.

## 1. Introduction

Grief over the loss of loved one is a commonly observed set of symptoms aroused by psychological and physiological reactions to the bereavement events [1]. Depression, an important indicator of bereavement-related mental health disorders [2], is closely associated with physical functioning [3], cognitive decline [4], and suicide tendency [5]. According to the stress process perspective, the wellbeing inequalities in ageing process could be contributed by stressors (i.e., negative life events) in earlier lives [6]. Child loss is one of the most traumatic life events for parents [7]. Bereaved parents often suffer negative emotions (e.g., sadness, detachment, guilt, and loss of control) [8]. However, to what extent the bereavement event could be a chronic life stressor may be affected by the pre-loss dependence, resilience with pre-loss acceptance of death [9], loss-oriented model or problem-focused model [10], and construction of meaning [11]. As shown in empirical studies, gender, lack of social support, poor financial or health problem, time since loss may be important predictors of the bereavement outcome [12]. In contemporary China, child loss is a rare event proportionally, but the sheer number is huge. According to the 1% of census data in 2005, about 1.6% of Chinese parents had experienced child loss [13]. It is estimated that child loss has impacted about one million families in China and is predicted to occur to 10 million families by 2035 [13]. Although the Chinese government has made substantial efforts, including providing economic subsidy, to alleviate the negative impacts of child loss for bereaved parents [14], the concern over the wellbeing of bereaved parents persists [13].

As suggested by the intergenerational system in context (ISC) model, macro level factors are influential in the process of parent-child ties [15]. Social-cultural contexts shape individual roles according to life schedules, and the socially expected role sequences and timing affect how parents and children are linked to each other [16,17]. Social timing, which emphasizes the incidence, duration, sequence of roles, and age-related expectations over the life span [16], may help to understand the child bereavement effect across societies. Therefore, this study proposes that the intergenerational influence of family issues on parental wellbeing could depend on its timing. The persistent intergenerational solidarity over the life course between children and parents could make the child loss event as a life-long struggling in China. Parents anticipate the support from children in later life to repay their investment in the childrearing, claimed as “Feedback” (*fanbu*) in Confucian norms [18,19]. In China, support from extended families is still the main form of old-age security for the older adults, especially in rural area [19,20]. After child loss, influential factors in coping with the stressful event, such as meaning reconstruction about life, alternative opportunities and available resources, are contingent on both parents and children’s life stages [14,21]. However, the existing studies have rarely examined the role of social timing in the child bereavement impact. Furthermore, there are socially structured gender-specific role expectations between children and parents across societies. As a result, to what extent parents can cope with the stress arising from child loss may be contingent on the child’s gender in some contexts. 

We situate our study in China, where traditional gender role expectations are still strongly held despite some changes. Using data from a nationally representative social survey, the China Health and Retirement Longitudinal Survey (CHARLS), we aim to fill the gaps in the literature on the heterogeneous influence of child bereavement on parental wellbeing by children’s life timing. Moreover, a gender and life course perspective enables us to clarify saliences of cultural reluctance in value changes in East Asian societies. This study goes beyond prevalent findings on the static occurrence effect, by disclosing the dynamic process of linked lives in the case of child loss over the life course. 

## 2. Literature Review

### 2.1. Child Loss and Parental Wellbeing

The death of a child is one of the most traumatic events for parents, which often has long-term devastating effects on parental wellbeing [7]. For example, previous studies have shown that bereaved parents in their early 50s still suffer from grief an average of nearly 18 years after child loss [22]. To examine the process, psychologists tend to focus on the parental grief process for a child’s death, and have developed theories such as the meaning reconstruction theory [11] and cognitive stress theory [23] to interpret parental grief. Meaning reconstruction theory [11] argues that the death of a child may demand parents reorganize personal identity and construct life purposes after child loss. Cognitive stress theory [23] takes an individualistic perspective, and contends that the way individuals react to illness and life tragedy affects the extent to which they are affected. These theories have shed important light on the understanding of the child bereavement effect by capturing snapshots of parental grief. What is missing from these perspectives is the exploration of parental grief over child loss from the perspective of socially constructed intergenerational linkage across both parents and children’s life stages, one of the theoretical arguments under the framework of life course perspective. 

According to the life course perspective, children and parents’ life chances are interconnected, which may last from birth into later life [24]. The strong tie between parents and children is one of the important sources of social integration, helping adults construct the meaning of life, expanding social connection, and maintaining social security [25]. Child loss leads to a declined sense of security and life meaning among bereaved parents due to the intergenerational tie interruption [26]. Moreover, the life course perspective points to the significance of sequences of life events in wellbeing. When life events deviate from normative life schedules, it may also be destructive for later life chances [17,27]. In this sense, the death of children disturbs the parents’ expected life course for both themselves and their lost children, which in turn tends to generate parental stress due to the failure in fulfilling the mission of being parents. Related to this, the child loss event may create new life strains or intensifies pre-existing strains, which further provoke a broad range of adverse life outcomes [6]. 

In previous studies, scholars have found consistent negative consequences to parents due to child loss, such as post-traumatic stress disorder (PTSD) [28], anxiety and depressive symptoms [22,29], a higher rate of chronic diseases [30], and a higher maternal mortality [31,32]. Despite that most studies have been conducted in Western societies, given the importance of family support in later life and the cultural values of support exchange between adult children and parents in East Asian societies [18], child loss may exert similar or even stronger detrimental effects to parents in such a context. Therefore, we hypothesize the following:

**Hypothesis** **1** **(H1).** *Child bereaved parents who have experienced child loss are more likely to report depression comparing to parents who have not suffered child loss*.

### 2.2. Social Timing of Child Loss and the Stress Process

Although parents may have a similarly strong level of attachment to their children in all life stages, norms associated meaning reconstruction after child loss, parents’ needs and resources in the stress process caused by child bereavement may vary according to individual’s social timing. By integrating the social timing and stress process, we build a dynamic theoretical framework to understand the impacts of social timing on intergenerational ties, as well as the interaction effects between individual agency, structural constraints and opportunities determined by contextual factors [33].

According to social timing argument from the life course perspective, the interdependent association between children and parents is not fixed but dynamic in the long term. There are life-cycle variations in intergenerational solidarity between children and parents [16,34]. The flow of social support between two generations responds to resources and the needs, differentiated by the life course status of parents and children [35,36]. When children are young, parents emotionally and financially invest in them and anticipate the children will repay the “debts” in later parental life stages, as suggested by the exchange theory [37]. In this process, parents play the role of protectors of children before they become adults [38] and strive to help their young adult children achieve successful life course transitions, such as finding a job, forming a partnership and parenthood which also mark parents’ own role fulfillment [39,40,41]. The social, emotional, and financial support often flows from parents to children in children’s childhood and young adulthood, and such parental support declines when children age and become independent [42]. However, when parents themselves gradually retreat from the labor market, they start to face constrained social network and life challenges caused by declining functional abilities [43]. In this stage, parents are not in fertile ages, tend to offer less and demand more from living children, and the intergenerational transfer flow is reversed from children to parents in parents’ later life [38]. Given that parental grief over child loss could be mainly aroused by the practical concern about support from children and life securities in later life, we hypothesize the following:

**Hypothesis** **2a** **(H2a).** *The death of an adult child in midlife or later when parents are at an advanced age may have more negative consequences on parental wellbeing compared with bereavement occurring when a child is younger*.

In contrast, according to cultural norms on children’s adulthood transitions, the death of a child in young adulthood may hurt parental role achievement for parents, and a much greater effort may be needed to recover from the death of a child in young adulthood than at other timing points. Therefore, if parental grief over child loss is mainly aroused by their own perceived parenthood fulfillment and lineage continuities in family roles, we propose a competing hypothesis of Hypothesis 2a:

**Hypothesis** **2b** **(H2b).** *The death of a child in young adulthood has more negative influences on parental wellbeing than other life stages*.

### 2.3. Gender, Child Loss, and Parental Depression

Gender difference in child bereavement studies is another important consideration in social contexts where son preference and traditional gender ideology are strong [29]. For people born before the period of industrialization and modernization, they tend to accept the traditional gendered norms of familism and associated expectations over children [44]. Gender ideology on children not only shapes the social interaction and sense of obligations, but also the degree of intimacy and exchange between parents and children [36] (p. 10). In such societies, women are socialized to be more family-oriented and pictured as kin-keepers [36] (p. 18) and are also expected to be important caregivers for children and parents-in-law in patrilocal co-residence [45]. However, such socially structured norms may have been weakened by the power of industrialization and modernization [44]. Female employment alters the traditionally male-centered familial dynamics [46]. Many studies have considered the gender differences in the support flow between children and parents in western industrialized societies, and it has been found that daughters actually provide more support to parents than sons [47]. The normative role expectations on children and the practiced roles of children in supporting parents may have differentiated the parental wellbeing responding to son and daughter’s death.

In East Asian societies, son preference culture supported by the Confucian family system is still strong, where male offspring are traditionally assumed to carry the family line and take care of old parents whereas daughters often had no right to claim their parents’ property after getting married [18,48,49]. Although such societies have undergone dramatic changes due to industrialization, changes in family expectations and obligations in contemporary East Asian countries are very limited [48]. The structured family roles and expectations, as well as the gendered social interaction between parents and children in East Asian societies, may differentiate how parents respond to the death of daughters and sons. However, despite the cultural expectations, a recent study has shown that daughters have a stronger filial piety towards parents and play a more important role in parents’ old-age support than sons [50]. It becomes more and more prevalent that old parents live with married daughters and are financially supported by daughters in urban China nowadays [49]. Therefore, patterns of health consequences by sex of children among bereaved parents may have been changed in recent decades due to the changing sex roles, although it is theoretically possible that parents may perceive child loss more from the perspective of traditional gender norms than from that of the practical caring they may receive from daughters. Previous studies in Chinese cultural context demonstrated both possibilities. In Taiwan, a study shows that the death of a son predicts mother’s suicide tendency [51], and another study demonstrates that only a son’s death is associated with the mother’s declined wellbeing [29]. However, research on the group of “*Shiduers*” (parents who have lost their only child) in China show that gender of the child does not differentiate the association between only-child death and their parents’ wellbeing [52], but it cannot be generalized to parents who have multiple children. Thus, in this study, we propose two competing hypotheses on child gender in the association between child loss and parental wellbeing. By normative perspective in terms of the traditional gender norms, we propose Hypothesis 3a; by the perspective of practiced roles in supporting parents, Hypothesis 3b is hypothesized.

**Hypothesis** **3a** **(H3a).** *A son’s death has more negative influences on parental wellbeing than that of a daughter*.

**Hypothesis** **3b** **(H3b).** *A daughter’s death has more negative influences on parental wellbeing than that of a son*.

## 3. Materials and Methods

### 3.1. Data and Sample

This study uses three waves of data from the China Health and Retirement Longitudinal Study (CHARLS), implemented by Peking University in 2011, 2013, and 2015 [53]. CHARLS is a nationally representative survey of respondents who are 45 years or older and their spouses, equivalent to the Health and Retirement Study (HRS) in terms of survey design. In this study, we restrict analytic samples to respondents aged over 45. The sample size is 16561 in waves 2011, among which 291 respondents reported complete information of child loss experience. In the wave 2011, 2130 respondents were deleted for missing values in depression score, and 77 respondents were deleted for missing values in childhood self-rated health, self-rated health, education and household registration status (i.e., rural or urban *hukou*). Then there are 14354 respondents kept in wave 2011, among which 234 respondents reported complete child loss information.

The analytic samples have been used in two different ways. In the first step, when we estimate the occurrence effect on parental wellbeing, the full sample is used. Then, in the second step, when we estimate the timing influences of child death on parental wellbeing, we focus on respondents who have experienced child death. All analyses use respondents followed for at least two waves across the three waves of the survey.

### 3.2. Variables and Measures

In this study, our dependent variable, depression, is measured by the 10-item measures from Center for Epidemiologic Depression Scale (CES-D) [54]. The 10-item CES-D comprises the following items: “I was bothered by things that don’t usually bother me; I had trouble keeping my mind on what I was doing; I felt depressed; I felt everything I did was an effort; I felt hopeful about the future; I felt fearful; My sleep was restless; I was happy; I felt lonely; I could not get going”. The possible responses to the CES-D questions include a four-scale metric (with points ranging from 0 to 3), representing the frequency of certain experiences in a week. The responses of positive emotions are reversely coded and are summed to create a scale with possible values from 0 to 30. Internal consistency reliabilities of the depression score as estimated by Cronbach’s alpha values range from 0.76 to 0.80 across the three waves. Depression is dichotomized and coded as 1 for those with depression score ≥10, and 0 for those with depression score <10. The validity of the dichotomized depression among Chinese population has been verified by Boey [55].

There are two key independent variables associated with child bereavement in this study. The first independent variable is “the occurrence of child loss”, measured by whether the main respondent has experienced the death of biological children. We then draw on information on the first deceased child, including gender, age of the deceased child and child’s life stage when the child died. In China, since people aged 18 and over are legally defined as adults and with age 40 indicating the start of midlife, we adopt ages 18 and 40 as cut-off points for young and middle adulthood respectively. Therefore, children’s life stage is also categorized into three types: before adulthood (aged 0–17), young adulthood (aged 18–39), middle and late adulthood (aged 40 and over).

Covariates include both time-constant variables and time-varying variables which may predict the bereavement outcomes [12]. Time-constant variables include gender (male = 1, female = 0), *hukou* (urban = 1, rural = 0), education attainment (1 = illiterate, 2 = primary education, 3 = junior high, 4 = senior high and higher), and self-rated health in childhood. Time-varying variables include time since child’s death, age, age-square, marital status (married = 1, unmarried = 0), logged family expenditure per capital, sum of living children, self-rated health (SRH), instrumental ability of daily living (IADL) (disabled = 1, abled = 0), and sum of chronic diseases.

### 3.3. Statistical Analysis

This study uses random-effects model for binary outcomes to estimate the influence of child bereavement on parental depression. Our independent variable, the child loss event, is treated as a time-constant life event occurring in the parents’ life history given the rare occurrence of such event. The random-effects model is a two-level model since we use panel data with each individual observed for multiple times across waves. Hence, every observation is nested with the individual, and Level 1 includes each observation whereas Level 2 includes each individual. The random effect model using panel data has advantages over ordinary least squares (OLS) in relaxing the independence assumption between independent variables and error term. As a result, coefficients estimated through panel data can account for inter-individual variances and have the advantage of being able to obtain more accurate inferences of model parameters, compared with estimates using cross-sectional data [56].

(1) $$\ln\left(\frac{P_{ij}}{\left(1-P_{ij}\right)}\right)=u+\gamma_{1}Cdeath_{i1}+\gamma_{2}z_{i2}\ldots +\gamma_{j}Z_{iq}+\beta_{i}X_{ij1}\ldots +\beta_{p}X_{ijp}+b_{1i}$$

(2) $$\ln\left(\frac{P_{ij}}{\left(1-P_{ij}\right)}\right)=u+\gamma_{1}Ctiming_{i1}+\gamma_{2}Cmale_{i2}+\gamma_{3}Ctiming_{i1}*Cmale_{i2}+\gamma_{4}z_{i4}\ldots +\gamma_{j}Z_{iq}+\beta_{i}X_{ij1}\ldots +\beta_{p}X_{ijp}+b_{1i}$$

From Equation (1) to Equation (2), $P_{ij}$ indicates the probability of reporting depression; Cdeath denotes exposure to child loss, Ctiming denotes the child’s life stage when parents experience child loss, Cmale denotes the gender of the deceased child and $Z_{i}$ denotes time-constant parental covariates (gender, *hukou*, self-rated health in childhood); $X_{ij}$ denotes time-varying variables (age, age-square, marital status, logged household expenditure per capital, time since the child’s death, sum of living children, self-rated health, IADL and sum of chronic diseases) for individuals across different times; $b_{1i}$ accounts for variability across individuals, is assumed to be independent of $X_{ij}$ and $Z_{i}$, drawn from a normal distribution.

## 4. Results

### 4.1. Descriptive Statistics

Table 1 shows the descriptive statistics of our key variables. In the baseline wave (2011), 1.67% of the 14,354 respondents have experienced child loss. Based on the retrospective information of the first deceased child, Table 1 shows the descriptive statistics of the analytic samples in our second step. The average age of the child at death is around 23, ranging from infancy to 65, and the average time since the child’s death is 24 years in 2011, ranging from 0 to 68 years. Among the deceased children, 38% died before adulthood, 40% died in young adulthood, and 22% died in middle and late adulthood. Of the deceased children, 63% are sons. The socio-demographic characteristics of the analytic sample in wave 1 have also been displayed in Table 1.

### 4.2. Analytic Results

Table 2 shows the influence of children’s death on parental depression. We have added covariates into models step by step. In the baseline model, we included the variables indicating social-demographic characteristics (age, gender, education, *hukou*), family structure and family resources (marital status, number of living children, logged household expenditure per capital). Then we further added the variables indicating health status into the unconstrained model. As shown in Models 1 and 2, the odds ratios of child loss are consistently larger than 1 and change from 1.628 to 1.677 after adding health relevant covariates. As shown in the unconstrained model (Model 2), the odds ratio of child loss is 1.677, meaning that parents in our sample who have experienced a child’s death are about 68% more likely to report depression than parents who have not lost a child. Therefore, hypothesis 1 on child loss effect is supported. Across Models 1 and 2, age, gender, education, marital status, *hukou*, and health indicators are significantly associated with the likelihood of reporting depression and serve as important covariates, so they are also included in the subsequent models to estimate the timing influences of child bereavement.

Table 3 shows how child bereavement effect varies by the child’s life stage. As shown in Model 3, parents whose children died before reaching adulthood do not report significantly different level of depression to those whose children died in midlife and later life. In contrast, parents of children that died in young adulthood are 2.488 times more likely to report depression than parents whose children died in middle adulthood or later. Therefore, compared with parents with children that died in young adulthood, parents with children whose death occurred after midlife or later are less likely to report depression. Thus the hypothesis on normative expectations on children’s adulthood transitions (H2b) is partially supported.

The interaction effect between gender and life stages of deceased children is shown in Model 4. It suggests that parents with a son that died in young adulthood are 9.295 times more likely to report depression than their counterparts with a deceased daughter. Therefore, the death of sons is more likely to predict parental depression than the death of daughters across the linked life courses, and thus the hypothesis on normative perspective in terms of the traditional gender norms (H3a) is supported.

## 5. Discussion

Our study makes important contributions to the influence of intergenerational ties to parents by examining the timing influences when facing the event of a child’s death. Despite previous studies having shown that the remorse and grief caused by child loss continues in parental life [7], few studies have examined its lasting effect on parental psychological distress and how such an effect varies by children’s life stages when they die. As shown in the previous study, social timing on a son’s adulthood transition indicated by marriage formation could be a significant stressor of Chinese parents [57]. The more severe influence on parents when the death occurs in children’s young adulthood than later adulthood suggests that parents’ grief over a child’s death is more about lineage continuities in family roles, rather than for practical concern about support from children and life securities in later life. This finding points to policy implications for government and social organizations in building up specific social welfare programs that should provide both resources and trained social workers to help people at different life stages to manage the stress associated with child loss.

Our findings on child gender difference regarding the death of a child show the continuation of strong son preference norms in a cultural context with patriarchal traditions. Although family responsibility and expectations have changed in East Asian societies in recent decades [48] and there is an emerging trend of increasing involvement of daughters in care giving [50], our results reveal the impacts of son preference culture continuity in the association between child loss and parental wellbeing in such a context. However, we expect such trends to change in the future when sibling numbers decrease, as impacted by the one-child policy. When more data become available, future studies might consider this from a cohort perspective to see whether gender deference disappears in more recent cohorts.

Despite of these contributions, our study has several limitations. First, the random-effects model is limited in ruling out bias contributed by unobserved time-constant confounders [58]. Biological factors, including genetic and personalities, could confound the association between a child’s death and parents’ subsequent life chances [59]. Family history of longevity as a proxy of genetic predisposition reduces the risk of mortality across generations [60,61]. Moreover, following SES-health gradient theory, unmeasured socioeconomic factors (e.g., living environments) are influential risk factors of mortality [62], and may also contribute to parental life chances. Second, we have only used information on the first biological deceased child reported in the baseline wave in estimating the exposure effect of child loss. Therefore, this study has not considered the cumulative effect of multiple experiences of child loss, due to lack of sufficient observations. Multiple stressors are more difficult to manage, and have more salient negative effects on mental health than a single stressor [6]. Third, a better way to examine the timing influences of child loss on parental life chances is individual-level longitudinal data across the whole adult life course, which could be informative to make convincing causal inferences [63].

Fourth, since this study has not considered the causes of child death, it is limited to demonstrate the heterogeneous effect of child bereavement contributed by the causes of death. The causes of death which may affect the pre-loss acceptance of death [9], may be an alternative explanation of the timing effect of child loss shown in this study. For example, death by suicide may be more common in young adulthood versus in childhood or later adulthood. Moreover, parents may be more reluctant to accept the death of a child in young adulthood occurred abruptly. Future studies may shed light on how causes of death may affect the bereavement outcomes through the pre-loss acceptance of death [9]. Lastly, since child loss is a rare event, a nationally representative dataset often has few such samples, which leads to a small sample size in our analysis. Future studies can focus on different groups of individuals with a qualitative approach in order to delve deeper into the parental grief process caused by a child loss event.

## 6. Conclusions

The death of a child is a traumatic life event for parents [7]. This study aims to reveal the heterogeneity among bereaved parents across their children’s life stages, and also to examine how child loss effect is shaped by social-cultural structured norms in terms of gender role and expectations. By drawing on retrospective information on children’s deaths, this study has the advantage of using random-effects models to examine the linked lives according to the case of the child loss event.

Our study yields three important findings. First, we provide empirical evidence on the negative impact of child loss experience on parental depression in China. This finding is consistent with the suggestions of linked lives in life course theory [16,24], as well as the empirical findings in developed contexts [22,29]. Despite of being unable to compare the degree of influence across contexts, child loss could have a particularly powerful outcome for parents in the Chinese sociocultural context, being literally described as a life tragedy in a Chinese saying, “the white-headed witness the death of the black headed” (*Bai Fa Ren Song Hei Fa Ren*). Second, we show that bereavement occurring in a child’s young adulthood is more likely to have a detrimental influence on parental psychological wellbeing than a death occurring in the child’s middle and late adulthood. It suggests that the unfulfillment of adult roles caused by the death of a young adult child has a more detrimental influence on the parental grief process than the death of an adult child in midlife or later, as young adulthood is key stage in the achievement of their adult children marital and family expectations [34]. By comparison, parental role expectations on children may not yet have been formed during their children’s childhood. Third, this study also reveals that the timing influences of child loss varies by gender of children. Parents who have experienced the death of a son in the child’s young adulthood are more likely to report depression than that of a daughter. This finding provides support to the normative perspective in terms of the gender norms in patriarchal societies, which is in line with traditional gender norms of familism, and consistent with Lee’s study in Taiwan [24]. Because sons carry the family line [18], a son’s death has a more detrimental and disruptive outcome for parents.

## Figures and Tables

**Table 1:** Statistics of analytic sample in wave 1.

Variables	Mean (SD)	Range	N
Exposure to child loss (%)	1.67	-	14,354
Age	59.16 (9.59)	45–101	14,354
Male (%)	47.7	-	14,354
Education (%)			14,354
Illiterate	26.3	-	14,354
Primary school	39.5	-	14,354
Middle school or higher	34.2	-	14,354
Urban *hukou* (%)	22.4	-	14,354
Married (%)	88.5	-	14,354
Logged expenditure per capital	8.67 (0.86)	0–11.61	14,354
Total number of living children	2.65 (1.33)	1–10	14,354
Self-rated health	2.03 (0.87)	1–4	14,354
Total number of chronic diseases	1.38 (1.39)	0–9	14,354
IADL (%)	12.1	-	14,354
Self-rated health in childhood	3.73 (1.07)	1–5	14,354
Information of the first deceased child			
Child’s age when the child died	23.36 (17.93)	0–65	234
Time since the child’s death	23.89 (17.44)	0–68	234
Child’s life stage when the child died (%)			
Before adulthood	37.6	-	234
Young adulthood	40.6	-	
Middle and late adulthood	21.8	-	
Son (%)	63.3	-	234

Notes: Data source is CHARLS in 2011; SD: Standard deviation.

**Table 2:** Random-effects logistic regression models predicting parental depression by child loss.

Variables	Model 1			Model 2		
	O.R.	95% CI	S.E.	O.R.	S.E.	95% CI
Death of a child (ref., No)	1.628 *	[1.106, 2.397]	0.321	1.677 **	0.298	[1.184, 2.374]
Age	1.126 ***	[1.069, 1.186]	0.03	1.048 +	0.025	[0.999, 1.099]
Age-square	0.999 ***	[0.998, 0.999]	0.00	0.999 **	0.00	[0.999, 0.999]
Male (ref., Female)	0.472 ***	[0.429, 0.519]	0.023	0.571 ***	0.025	[0.524, 0.621]
Married (ref., unmarried)	0.534 ***	[0.466, 0.610]	0.036	0.540 ***	0.034	[0.478, 0.611]
Urban (ref., Rural)	0.502 ***	[0.444, 0.567]	0.031	0.530 ***	0.03	[0.474, 0.592]
Education (ref., Illiterate)						
Primary school	0.887 *	[0.793, 0.993]	0.051	0.903 *	0.047	[0.816, 0.999]
Middle school or higher	0.464 ***	[0.405, 0.530]	0.032	0.567 ***	0.035	[0.503, 0.640]
Logged household expenditure	1.028	[0.994, 1.064]	0.018	1.002	0.017	[0.969, 1.035]
Sum of living children	1.106 ***	[1.068, 1.146]	0.02	1.058 ***	0.018	[1.024, 1.093]
Self-rated health in childhood				0.891 ***	0.016	[0.859, 0.923]
Self-rated health				0.464 ***	0.011	[0.443, 0.485]
IADL (ref. Abled)				2.484 ***	0.125	[2.252, 2.741]
Sum of chronic diseases				1.282 ***	0.017	[1.248, 1.316]
BIC	40,291.693			37,558.56		
Log-likelihood	−20,083.1			−18,695.5		
N	35,013			35,013		

Notes: *p* < 0.1 +; *p* < 0.05 *, *p* < 0.01 **, *p* < 0.001 ***; two tailed tests.

**Table 3:** Random-effects logistic regression models predicting parental depression by life course of deceased child.

Variables	Model 3			Model 4		
	O.R.	95% CI	S.E.	O.R.	95% CI	S.E.
Child life course (ref., Midlife and later)						
Before adulthood	3.36	[0.598, 18.87]	2.958	3.001	[0.388, 23.309]	3.132
Young adulthood	3.488 *	[1.174, 10.358]	1.937	0.659	[0.122, 3.565]	0.568
Son (ref., Daughter)	0.534	[0.249, 1.141]	0.207	0.277	[0.056, 1.367]	0.226
Interaction items						
Child life course # Child’s gender						
Before adulthood * Son				0.608	[0.089, 4.175]	0.598
Young adulthood * Son				10.295 *	[1.409, 75.226]	10.447
BIC	596.507			595.635		
Log-likelihood	−240.3			−233.7		
N	447			447		

Notes: *p* < 0.1 +; *p* < 0.05 *, *p* < 0.01 **, *p* < 0.001 ***; two tailed tests. Covariates including age, age-squared, gender, marital status, education, logged family expenditure, number of living children, self-rated health in childhood, self-rated health, IADL, chronic diseases are controlled.
